# Supplementary material for: A cellular and molecular analysis of SoxB-driven neurogenesis in a cnidarian
Source: eLife. 2022 May 24;11:e78793. doi: 10.7554/eLife.78793 (PMC9173746; doi:10.7554/eLife.78793)
Supplement: Supplementary file 1. [file elife-78793-supp1.docx]

| Piwi1^+^ i-cells | Vs | Control (GFP^-^) |
| --- | --- | --- |
| SoxB2 NPs | **Vs** | Control (GFP^-^) |
| GFP^high^ neurons | **Vs** | Control (GFP^-^) |
| GFP^low^ neurons | **Vs** | Control (GFP^-^) |
| Piwi1^+^ i-cells | **Vs** | SoxB2 NPs |
| SoxB2 NPs | **Vs** | Piwi1**^+^** i-cells |
| GFP^high^ neurons | **Vs** | GFP^low^ neurons |
| GFP^low^ neurons | **Vs** | GFP^high^ neurons |
| SoxB2 NPs | **Vs** | GFP^high^ neurons |
| GFP^high^ neurons | **Vs** | SoxB2 NPs |
| SoxB2 NPs | **Vs** | GFP^high^ neurons |
| GFP^low^ neurons | **Vs** | SoxB2 NPs |
