# Supplementary figures and images for: A cellular and molecular analysis of SoxB-driven neurogenesis in a cnidarian
Source: eLife. 2022 May 24;11:e78793. doi: 10.7554/eLife.78793 (PMC9173746; doi:10.7554/eLife.78793)

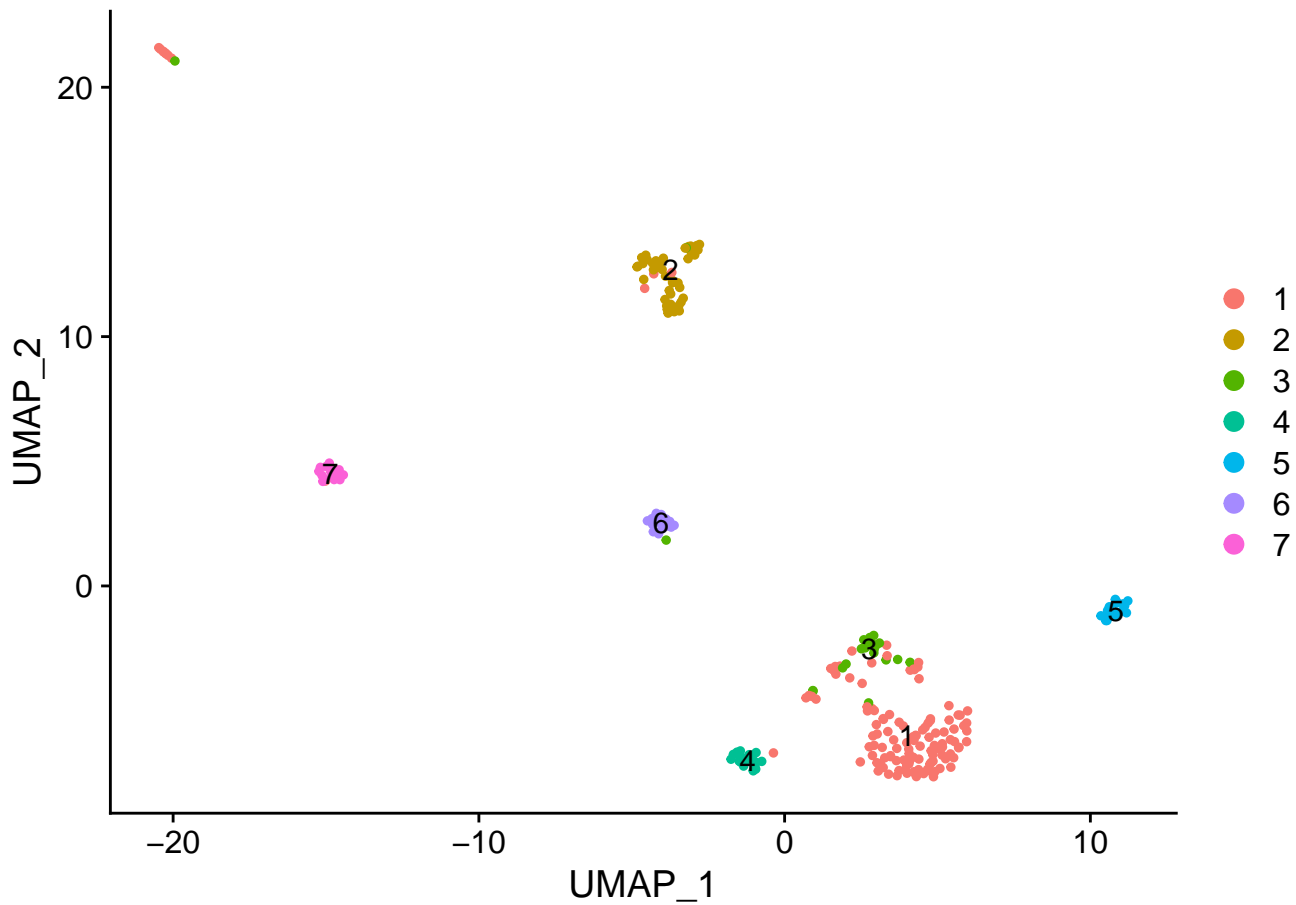

Supplement: Supplementary file 3. [file elife-78793-supp3.zip › Supplemental File 2/subcluster_7/UMAP_cor_cluster7_subclusters.pdf]

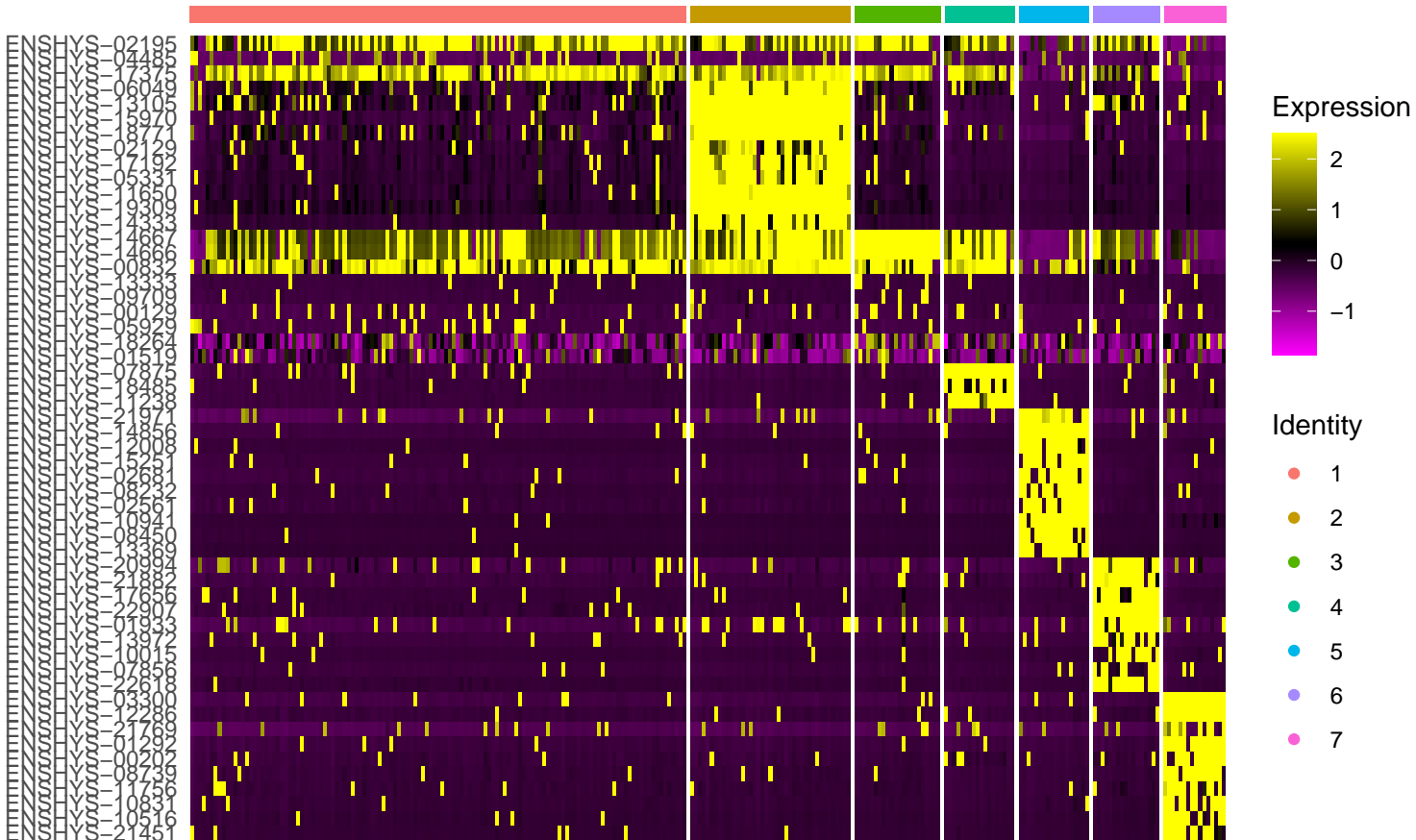

Supplement: Supplementary file 3. [file elife-78793-supp3.zip › Supplemental File 2/subcluster_7/heatmap_top10_markers_cluster7.pdf]

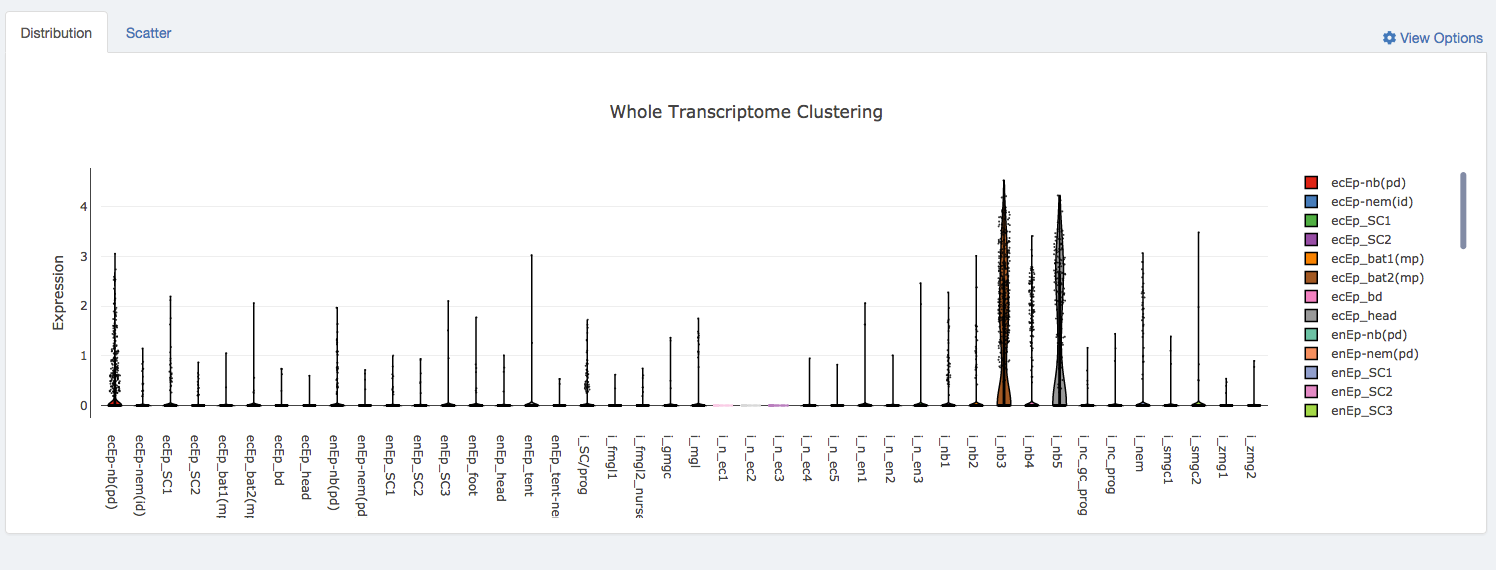

Supplement: Supplementary file 3. [file elife-78793-supp3.zip › Supplemental File 2/subcluster_7/proline_rich_Hydra_1.tiff]

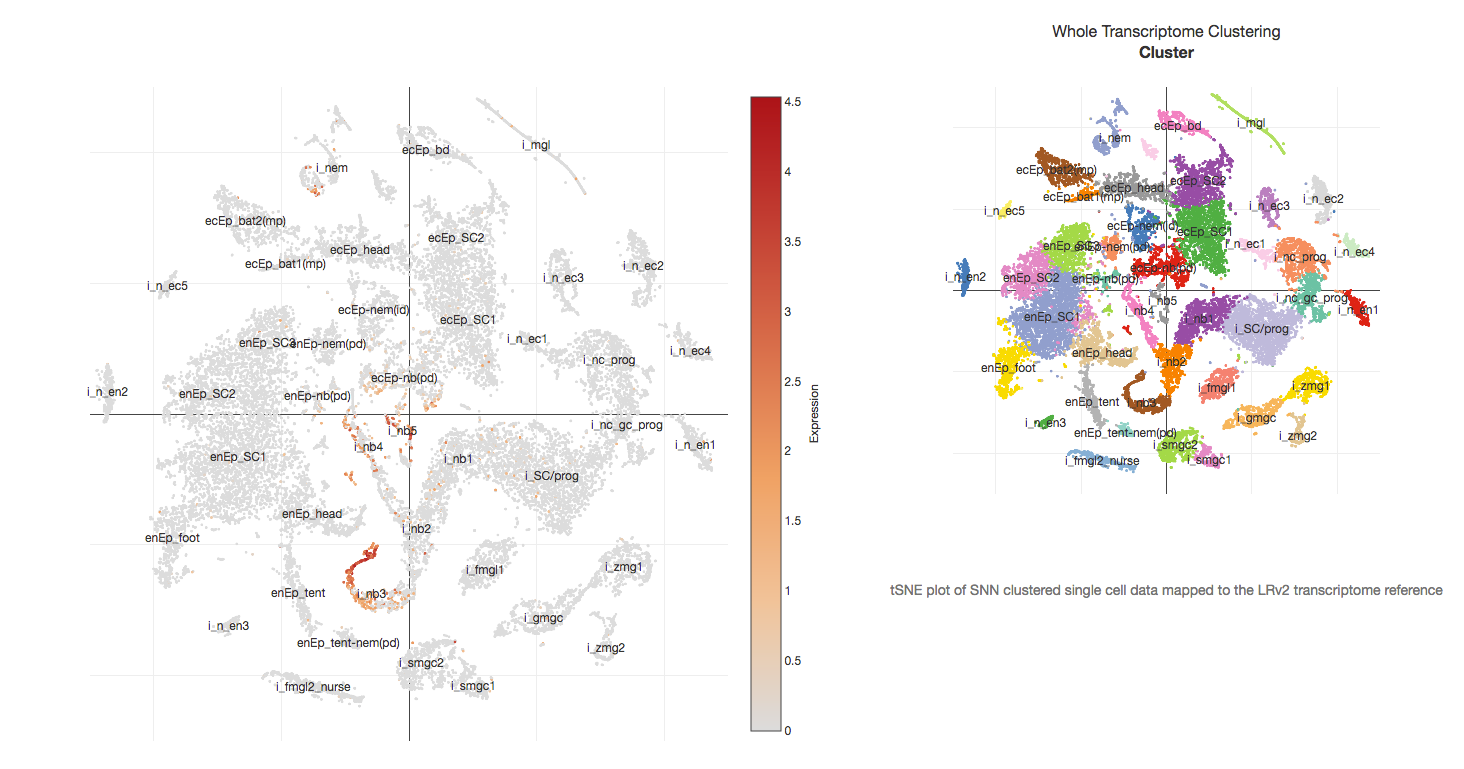

Supplement: Supplementary file 3. [file elife-78793-supp3.zip › Supplemental File 2/subcluster_7/proline_rich_Hydra_2.tiff]

**ENSHYS-02209**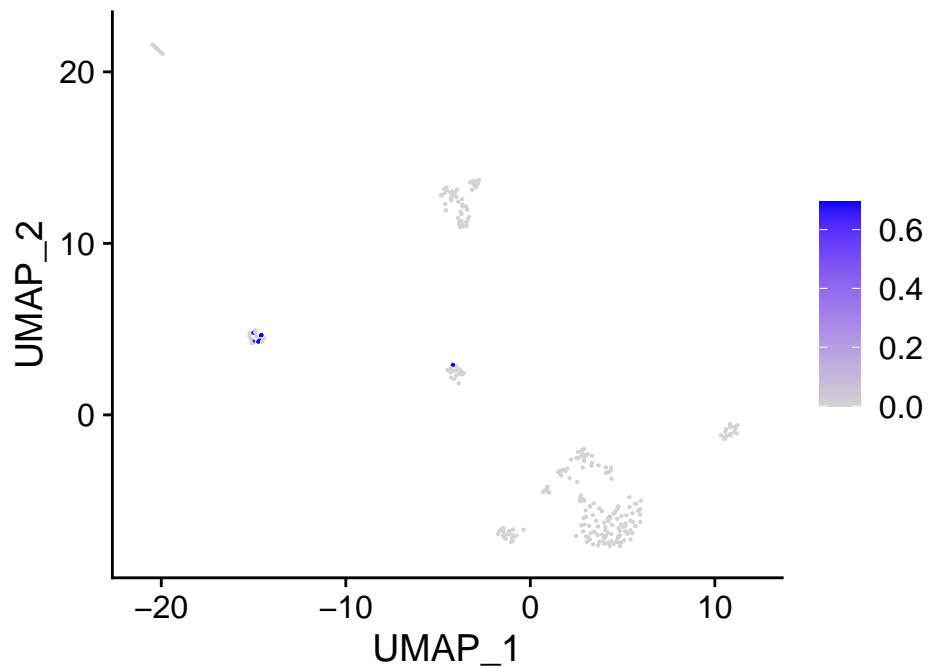**ENSHYS-01775**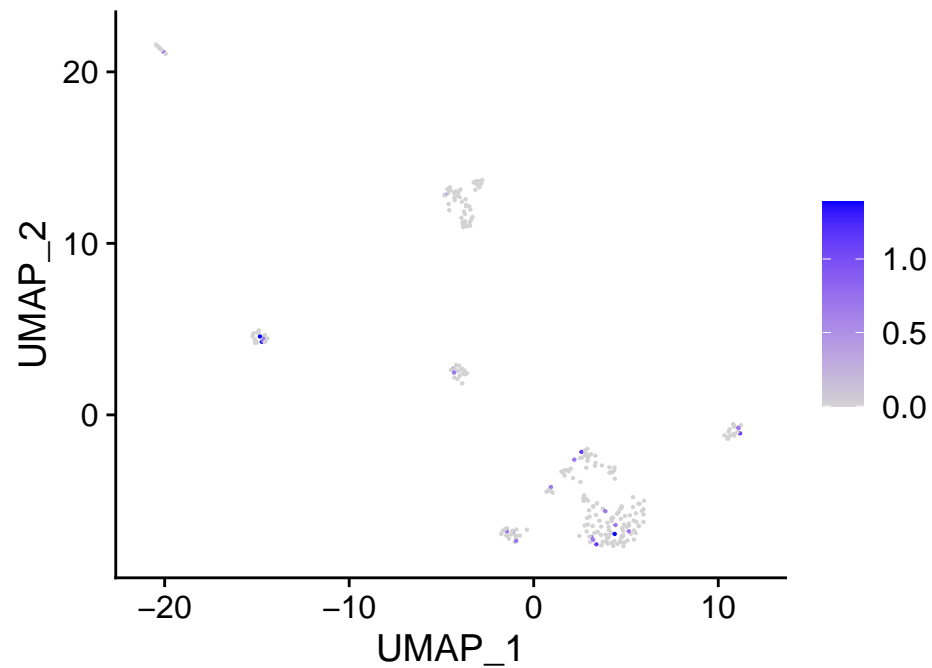**ENSHYS-18955**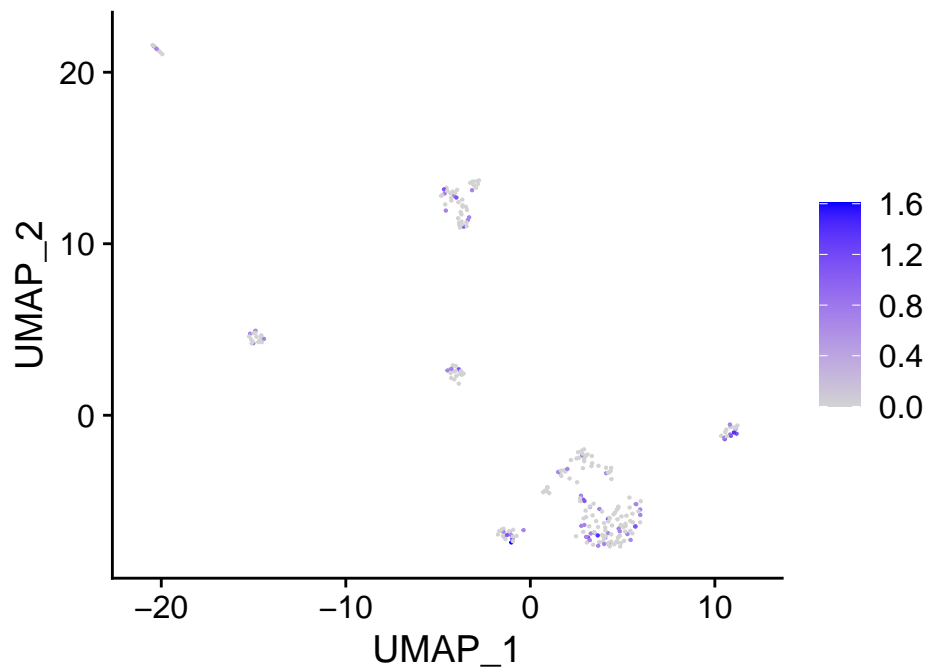**ENSHYS-19760**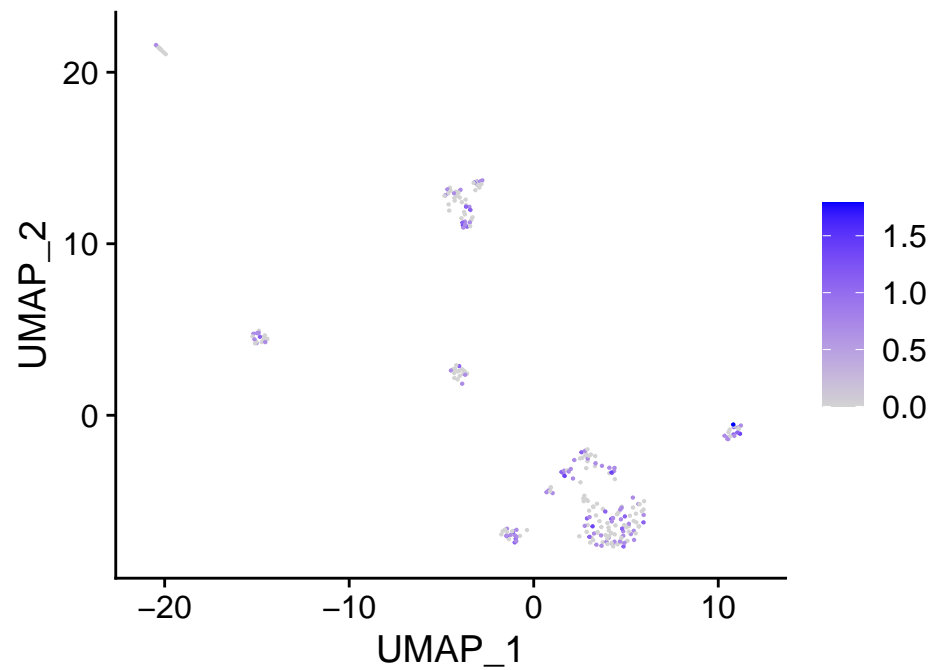

Supplement: Supplementary file 3. [file elife-78793-supp3.zip › Supplemental File 2/subcluster_7/subclusters_cluster_7_markers.pdf]

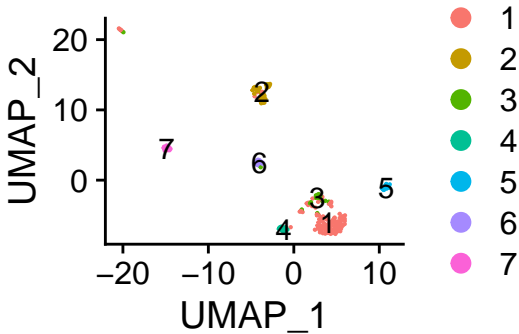

Supplement: Supplementary file 3. [file elife-78793-supp3.zip › Supplemental File 2/subcluster_7/subclusters_cluster_7_umap_dense.pdf]

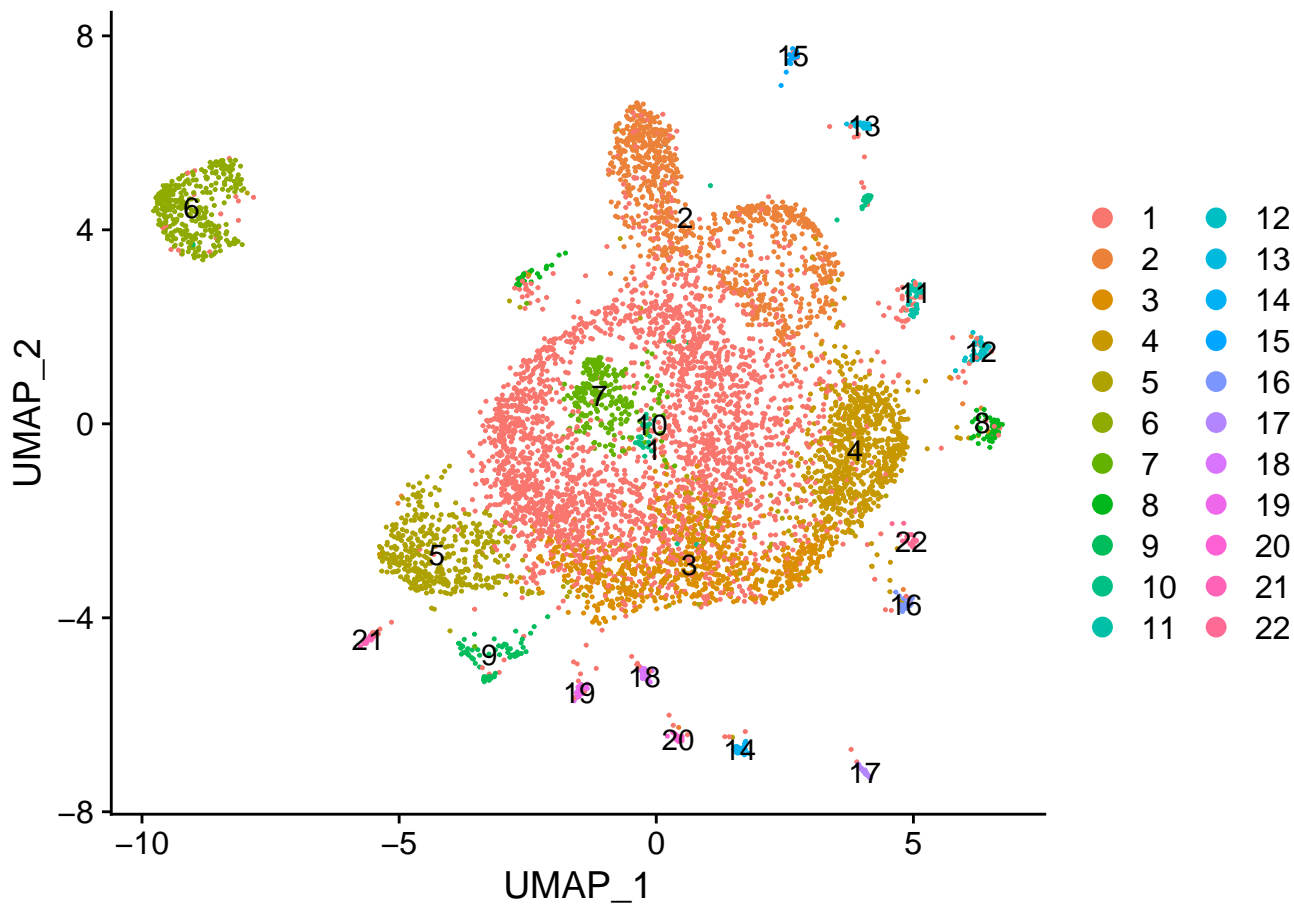

Supplement: Supplementary file 3. [file elife-78793-supp3.zip › Supplemental File 2/all_cells/UMAP_cor_cluster_number.pdf]

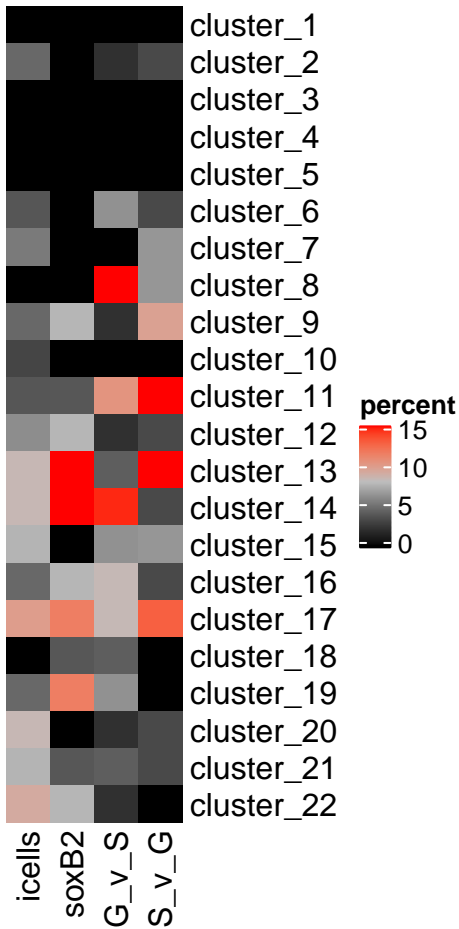

Supplement: Supplementary file 3. [file elife-78793-supp3.zip › Supplemental File 2/all_cells/DEG_overlap_heatmap.pdf]

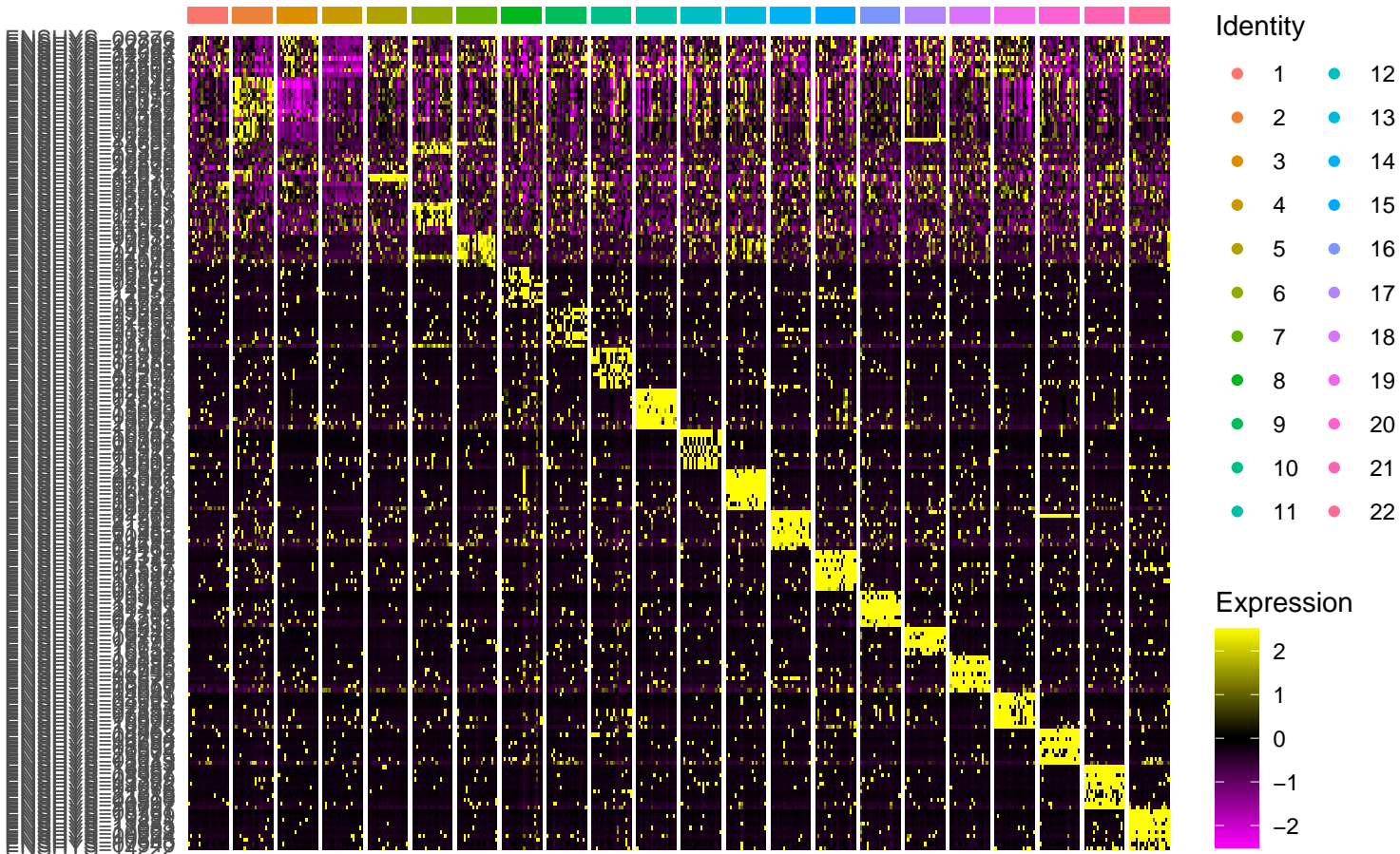

Supplement: Supplementary file 3. [file elife-78793-supp3.zip › Supplemental File 2/all_cells/heatmap_top10_markers.pdf]

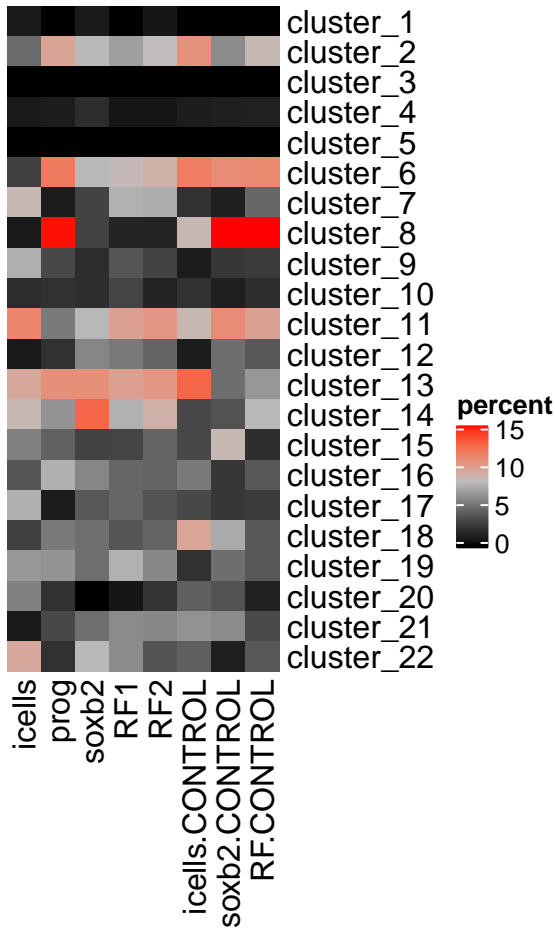

Supplement: Supplementary file 3. [file elife-78793-supp3.zip › Supplemental File 2/all_cells/RNA_quant_overlap_heatmap.pdf]
